# Characterization of the complete chloroplast genome of *Alsophila spinulosa*, an endangered species endemic to China

**DOI:** 10.1080/23802359.2020.1772142

**Published:** 2020-06-01

**Authors:** Yiyong Ma, Peng Jiao, Zhuo Qi, Zhenzhong Jiang, Shuyan Guan

**Affiliations:** Jilin Agricultural University, Changchun, China

**Keywords:** *Alsophila spinulosa*, endangered species, chloroplast genome

## Abstract

Tree fern *Alsophila spinulosa* is an endangered relic plant in the world. It is currently on the International Union for Conservation of Nature (IUCN) red list of threatened species. In this study, we first assembled the complete chloroplast (cp) genome of *A. spinulosa* by Illumina paired-end reads data. The whole genome was 156,661 bp, consisting of a pair of inverted repeats of 24,364 bp, large single copy region and a small single copy region (70,352 and 21,624 bp in length, respectively). The cp genome contained 133 genes, including 92 protein-coding genes, 33 trRNA genes, and eight rRNA genes. The overall GC content of the whole genome was 40.4%. A neighbour-joining phylogenetic analysis demonstrated a close relationship between *A. spinulosa* and *Cystoathyrium chinense* Ching.

*Alsophila spinulosa* is the only relict tree fems since the Jurassic Period, which has been endangered by habitat fragmentation (Tryon and Tryon 1982). The community of *A. spinulosa* has been restored with the increase of conservation intensity, but recent studies have found that most Cyatheaceae communities are experiencing decline and seedling reduction, some scholars also believe that the appearance of this phenomenon may be related to human activities (Kiran et al. 2012).

In this study, *A. spinulosa* was sampled from the Endangered Species Reserve of Jilin Agricultural University Changchun County, China (125°19′E, 43°43′N). A voucher specimen (JN20200501) was deposited in the Herbarium of the Plant Biotechnology Center of Jilin Agricultural University, Changchun, China.

The present study is the first time to assemble and characterize the complete chloroplast genome for *A. spinulosa* (GenBank: NC_012818.1) from hight-hrough-put sequencing data. The existing chloroplast Genome sequence of ginkgo biloba was downloaded from the National Center for Biotechnology Information’s Organelle Genome Resources database (nc_016986.1) as the reference sequence, and the chloroplast Genome of *A. spinulosa* was assembled using SPAdes v3.6.0 software (Bankevich et al. 2012). The default setting of parameters was adopted. Sequence annotation first confirmed the availability and boundary of genes by blastn comparison directly through the protein-coding sequence of the proximal species. Then, the genes in the chloroplast genome were annotated by online tool DOGMA (http://dogma.ccbb.utexas.edu/) with default parameters, and the genes were functionally annotated by combining with NR (http://www.ncbi.nlm.nih.gov/) database (Lohse et al. 2013). TRNA was annotated using the trnascan-se online site. Using RNAmmer 1.2 Server (http://www.cbs.dtu.dk/services/RNAmmer/) rRNA for comments. The chloroplast genome of ginkgo biloba was mapped using OGDRAW (http://OGDRAW.Mpimp-golm.mpg.DE/cgi-bin/OGDRAW.Pl) software (Asaf et al. 2017).

The complete cp-DNA of *A. spinulosa* was a circular molecule 156,661 bp in length, comprising a large single copy (LSC) region of 70,352 bp and a small single copy (SSC) region of 21,624 bp, separated by two inverted repeat regions (IRs) of 24,364 bp. It contained 133genes, including 92 protein-coding genes, 8 ribosomal RNA genes, and 33 tRNA genes. The phylogenetic tree reveals all the species of Pteridophyta formed a monophyletic clade with high-resolution value and *Cystoathyrium chinense* Ching is most related to *A. spinulosa* (Figure 1).

**Figure 1.**
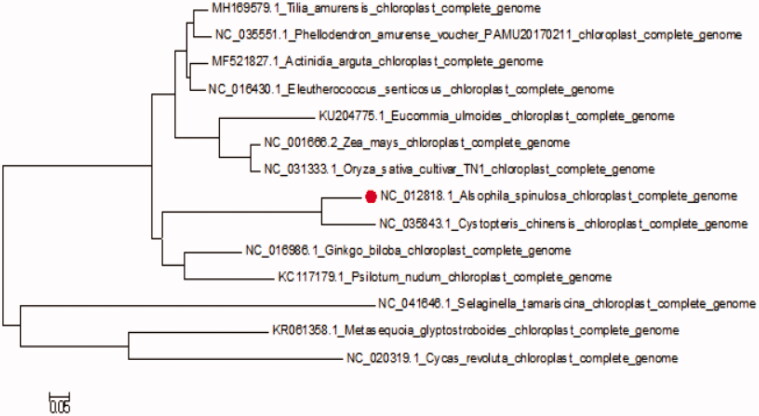
The phylogenetic tree based on 14 complete plastid genome sequences.

## Data Availability

The data that support the findings of this study are openly available in figshare at 10.6084/m9.figshare.12250268.
